# Digital Transformation and Cybersecurity Challenges for Businesses Resilience: Issues and Recommendations

**DOI:** 10.3390/s23156666

**Published:** 2023-07-25

**Authors:** Saqib Saeed, Salha A. Altamimi, Norah A. Alkayyal, Ebtisam Alshehri, Dina A. Alabbad

**Affiliations:** 1 Saudi Aramco Cybersecurity Chair, Department of Computer Information Systems, College of Computer Science and Information Technology, Imam Abdulrahman Bin Faisal University, P.O. Box 1982, Dammam 31441, Saudi Arabia; 2 Department of Computer Information Systems, College of Computer Science and Information Technology, Imam Abdulrahman Bin Faisal University, P.O. Box 1982, Dammam 31441, Saudi Arabia2230500089@iau.edu.sa (N.A.A.);; 3 Saudi Aramco Cybersecurity Chair, Department of Computer Engineering, College of Computer Science and Information Technology, Imam Abdulrahman Bin Faisal University, P.O. Box 1982, Dammam 31441, Saudi Arabia

**Keywords:** digital transformation, cybersecurity, information technology

## Abstract

This systematic literature review explores the digital transformation (DT) and cybersecurity implications for achieving business resilience. DT involves transitioning organizational processes to IT solutions, which can result in significant changes across various aspects of an organization. However, emerging technologies such as artificial intelligence, big data and analytics, blockchain, and cloud computing drive digital transformation worldwide while increasing cybersecurity risks for businesses undergoing this process. This literature survey article highlights the importance of comprehensive knowledge of cybersecurity threats during DT implementation to prevent interruptions due to malicious activities or unauthorized access by attackers aiming at sensitive information alteration, destruction, or extortion from users. Cybersecurity is essential to DT as it protects digital assets from cyber threats. We conducted a systematic literature review using the PRISMA methodology in this research. Our literature review found that DT has increased efficiency and productivity but poses new challenges related to cybersecurity risks, such as data breaches and cyber-attacks. We conclude by discussing future vulnerabilities associated with DT implementation and provide recommendations on how organizations can mitigate these risks through effective cybersecurity measures. The paper recommends a staged cybersecurity readiness framework for business organizations to be prepared to pursue digital transformation.

## 1. Introduction

Digital transformation refers to adopting digital solutions in the business processes of organizations, which can result in significant changes in their business operations. Such modification can impact various aspects of an organization, for instance, user experience, business processes, target markets, customers, customer relationships, and even diverse cultural implications. The accelerated technology adoption by business organizations during the COVID-19 pandemic also resulted in many abrupt challenges [1]. Emerging technologies such as artificial intelligence, big data and analytics, blockchain, cloud computing, the Internet of Things, and the industrial Internet of Things are critical enablers for digital transformation. Due to extensive benefits, businesses are accelerating the digital transformation drive. Still, cybersecurity has grown into a significant challenge for companies, and to gain business continuity, organizations need to secure their digital transformation tools and artifacts. Therefore, it is crucial for organizations undergoing DT adoption to prioritize cybersecurity measures and ensure that their systems are secure from potential threats [2,3].

Cybercriminals may take advantage of vulnerabilities in digital technologies; therefore, organizations must ensure that technological solutions are secure from digital attacks. Cybersecurity can be achieved by implementing encryption, authentication, and access control measures to protect data and networks from unauthorized access or malicious activities. Additionally, organizations should consider investing in cyber insurance policies that can provide financial protection against losses due to a successful attack on their systems. Another critical issue is to raise awareness among employees regarding cybersecurity attacks, as higher awareness results in dependable information security behavior [4,5]. Cyber-attacks have drastically escalated; therefore, business organizations must understand cybersecurity threats and how best to mitigate them comprehensively. These attacks usually aim to assess, change, or destroy sensitive information; extort monetary benefits from users; or interrupt normal business processes. Cybersecurity involves techniques to protect computers and networks from unauthorized access and malicious activities such as data theft and destruction.

Cybersecurity costs and cybercrimes are exhibiting an increasing trend globally [6]. Haislip et al. [7] highlighted that the economic cost of cybersecurity breaches is underestimated, as it is not only limited to the targeted form; they spill over to the industry concerned through negative returns and higher insurance costs. Garg [8] has highlighted seven critical benefits of investing in cybersecurity to motivate organizations in making cybersecurity investments. These include protecting intellectual property, better meeting customer requirements, minimizing customer turnover, branding secure products, joining secure vendors in an integrated network, company reputation, and minimizing collateral damage in the industry. Lee [9] has presented a risk management framework focusing on continuously improving cybersecurity practices and cost–benefit analysis for cybersecurity investments. Many organizations use the National Institute for Standards and Technology (NIST) Cybersecurity Framework for cybersecurity risk management; however, the standard lacks a cost–benefit analysis. The Gordon–Loeb model has been proposed to identify which tier of NIST is more effective for a particular organization in terms of cost–benefit study [10]. Krutilla et al. [11] enhanced the Gordon–Loeb model by considering the depreciation cost of cybersecurity assets, which can impact the cost–benefit analysis of cybersecurity initiatives. Simon and Omar [12] highlighted that companies may be affected by cybersecurity risks via cybersecurity attacks on their supply chain partners, so they maintain that cybersecurity investments need to consider both coordinated and uncoordinated attacks. Uddin et al. [13] highlighted that cybersecurity weaknesses impact organizational growth and performance, and, especially for the banking sector, operational risks have increased due to cybersecurity threats. Curti et al. [14] highlighted that cybersecurity attacks are on the rise in the governmental sector, and to mitigate these threats, governments are increasing governmental operating costs and overall financing costs.

In this paper, we have conducted a systematic literature review that documents how digital transformation has changed the business sector and the implications of cybersecurity for digital transformation. We have investigated the papers published during 2019–2023 using PRISMA guidelines for conducting a literature review. We have proposed a cybersecurity readiness framework for business organizations pursuing digital transformation. The findings of this paper will help business organizations, practitioners, and researchers to grasp the state of the art in this domain and will form the basis for further research.

This paper is organized as follows: Section 2 outlines the methodology adopted to conduct the survey, and Section 3 discusses the literature in detail. Section 4 provides a discussion, and a conclusion is offered in Section 5.

## 2. Materials and Methods

**Figure 1:**
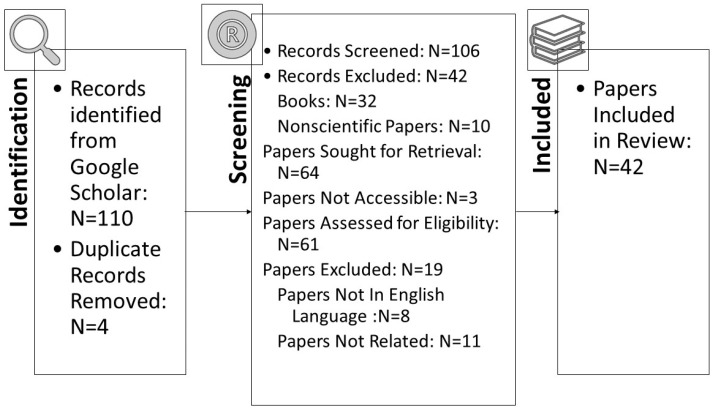
Prisma diagram for our systematic literature review.

In this section, we explain the methodology. We did a systematic literature review using the PRISMA guidelines [15]. As shown in Figure 1, we used the Google Scholar database. Primary studies were extracted using specific keywords in search criteria. Keywords were chosen to facilitate the generation of research articles relevant to our topic. The search terms used were (business transformation) AND (security), (digital transformation) AND (cybersecurity), (digital transformation) AND (cyber security), (digital transformation) AND (protection), and (digitization) AND (security). To refine our search results, we used the following inclusion criteria:

- The paper should be relevant to digital business and cybersecurity.
- The paper is published between 2019–2023.

Additionally, the following exclusion criteria were applied to search results:

- The papers are not written in the English language.
- The paper is not related to cybersecurity and digital transformation.
- The paper is a review paper.

All Google Scholar results were checked for compliance with these criteria. The process of identifying the extracted studies went through the quality assessment stage, starting with a quick scan of the title and the language of the paper (English or not). Secondly, it was also ensured that these papers are related to and relevant to our research. Figure 1 shows the number of final papers that were selected after going through these stages.

**Figure 2:**
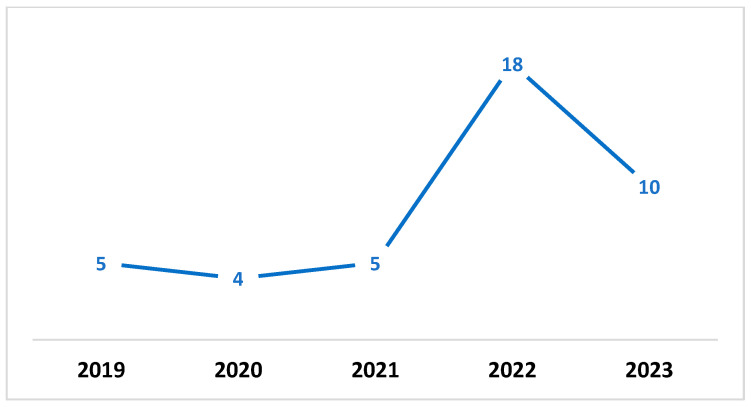
Year-wise publication history.

As highlighted in Figure 1, digital transformation and cybersecurity are widely researched, and our final analysis included forty-two papers. Figure 2 highlights the year-wise publication history.

## 3. Results

In this section, we highlight the findings of downloaded papers.

### 3.1. Financial Sector

The financial sector is a critical component of an economy, and there have been many empirical studies in different geographical contexts. For example, Al-Alawi and Al-Bassam conducted empirical research in Bahrain and found that financial institutions are exposed to online identity theft, computer system damage, and hacking attempts resulting in operational disturbances [16]. Similarly, Hasan and Al-Ramadan [17] conducted an empirical study with bank customers in Iraq and found that although banks adopt significant security measures, some customers are still skeptical about online banking. In another study, Joveda et al. [18] investigated the banking sector in Bangladesh. They highlighted developing a cybersecurity system for identifying money laundering transactions that negatively impact economic development. There is a vast potential in modern technologies to support the financial sector. Almudaires and Almaiah [19] outlined major threats to credit card companies and associated solutions for credit card companies to improve their cybersecurity implementation. Smith and Dhillon [20] highlighted that blockchain is a crucial technology to minimize security threats in financial transactions; however, there is a need for rigorous analysis of blockchain implementation in the financial sector. Similarly, Kuzmenko et al. [21] used machine learning models to analyze large volumes of financial data to identify potential threats at an early stage.

Rodrigues et al. [22] developed a decision-support model for incorporating artificial intelligence (AI), digital transformation, and cybersecurity into the banking sector while ensuring data security is not compromised. The authors found that traditional banks are under pressure from their stakeholders to adapt to new technologies, and they also need to ensure that any potential data breaches or other security issues do not compromise their reputation. The authors used cognitive mapping and the decision-making trial and evaluation laboratory method to address this complex issue with an expert panel in group sessions. This resulted in a realistic framework for making decisions regarding AI implementation in the banking industry while ensuring data security is not compromised. The study developed a multi-stakeholder cognition-driven framework using cognitive mapping combined with DEMATEL methodology. This approach allowed them to identify critical factors affecting AI adoption within banks, such as customer trust toward technology-based services offered by banks; regulatory compliance requirements; and availability of a skilled workforce, which were then ranked based on their relative importance using DEMATEL analysis. 

Similarly, Fedorov et al. [23] highlighted how cognitive technologies could ensure data security when using biometric identification technology in remote banking transactions. The article discussed how digital transformation and biometric identification would impact financial services in Russia. It emphasized that advanced security measures are necessary for protecting sensitive customer data during these transactions. The proposed solution is through cognitive technologies focused on human intellectual abilities as one direction for ensuring information security within this context.

Another research study by Patil and Bharath [24] investigated technological advancements in the financial sector. The study’s findings showed that Fintech has improved businesses, and investors have more confidence in the technology. They also presented new technologies adopted by Fintech and their associated issues. The effect of financial technology was positive on the factors of trust and business authorization. Traditional finance has noticed the most important critical issues, such as the risks of fraud and low performance, and differences and limitations have been encountered. The research was conducted on a limited sample of approximately 160.

Moreover, Rãƒdulescu et al. [25] explained the risks associated with digitalization regarding economic development and ensuring social and information security. They highlighted that digitalization significantly impacts economic growth, social inclusion, and sustainable development. However, it also introduces new vulnerabilities that can lead to cyber-attacks and require smart controls to prevent them. The authors suggested that technology experts and other stakeholders should be involved in assessing these risks as they can grow and become more complex over time. Risk managers must develop a comprehensive strategy that includes mitigation and risk transfer solutions, prioritizing which IT security options best mitigate the organization’s risk.

Moreover, international cooperation is essential to combat cybercrime due to the evolving global crime and terrorist threats associated with digital transformation. Finally, it highlighted the growing importance of information technology in business development, human relations, and communication between people and governments. Digital risk management should therefore be a priority for all involved stakeholders.

### 3.2. Health Sector

Cybersecurity in the health sector deals with patient data privacy [26] and the security of medical devices [27,28,29,30]. A secure digital transformation drive can help improve health organizations’ organizational governance [31,32,33]. Garcia-Perez et al. [34] discussed how the digital transformation of healthcare systems must be managed effectively from a cybersecurity perspective. This paper analyzed data from higher management in the UK during the COVID-19 pandemic. According to their findings, a balanced foundation that considers cybersecurity knowledge development, uncertainty management, and the sector’s high systematic and organizational interdependence that has implications for research and management practices is essential for digital resilience and sustainability efforts in the health sector.

On the other hand, Paul et al. [35] discussed the use of digital technology in the healthcare sector and highlighted privacy and security issues related to these technologies. This study examined how digitization is transforming the healthcare sector, its impact on patient care, and opportunities for new business models with Industry 4.0 and business intelligence approaches. The rise in chronic diseases and the current pandemic have increased the need for person-centered care that encourages individuals to be involved in their health care. Digital solutions such as biosensors and software are being introduced to meet the growing need for on-demand healthcare services. Big data analytics have also significantly impacted healthcare organizations by providing access to decades of stored data, which serves as evidence-based medicine for better decision-making when treating patients while ensuring patient privacy remains protected. There are many ways to address security and privacy concerns related to digitalization in healthcare. It covers various solutions such as mutual authentication, key agreement, lightweight cryptography, blockchain-based solutions, etc., which can help ensure the secure handling of medical data. The authors also suggest developing management programs for medical equipment and investigating how patient engagement can impact privacy and security measures. Finally, they recommend further research on regulations regarding privacy and security in the healthcare sector and exploring the role of artificial intelligence (AI) and blockchain technology in improving healthcare outcomes while maintaining data safety. The adoption of cloud-based technology is also discussed as a potential solution for better patient data archiving and usage, lower storage costs, quicker innovation cycles, more straightforward collaboration, and increased telemedicine possibilities.

Nwaiwu and Mbelu [36] highlighted that the General Data Protection Regulation GDPR is essential for businesses and governments to comply with to track and monitor people’s health, develop business models, and discover market opportunities. Statistics show that Europe has recorded 1.92 million confirmed COVID-19 cases and contact tracing with personal data is necessary to limit and contain the spread of the virus.

Maleh and Mellal [37] provided insights into how digital transformation and cybersecurity are impacted by COVID-19 proliferation. The author discussed how COVID-19 has accelerated digital transformation trends such as cloud computing, the IoTs explosion, and big data accumulation while also increasing cyber-attacks related to personal data protection. The three main categories of challenges faced by cybersecurity departments during and after the pandemic are resilience against cyber attackers exploiting crises such as phishing or ransomware; recovery by ensuring secure pre-COVID-19 working methods upon return to the office; and adapting a technology roadmap for new realities while meeting business needs and customer expectations in digital transformation projects.

### 3.3. Governmental Sector

Digital transformation in governmental organizations is adopted all over the world, such as in Bahrain [38], the UK [39], and Saudi Arabia [40]; however, the adoption speed is not uniform. Al Shobaki et al. [41] investigated how digital transformation affects cybersecurity practices within the Ministry of Interior and National Security in Palestine. The researchers used a descriptive-analytical approach with a questionnaire as their primary research tool. They found a statistically significant correlation between all digital transformation dimensions and the ministry’s cybersecurity practices. Additionally, certain organizational factors were found to have a powerful impact on these practices. For example, effective data exchange among different departments was identified as crucial for maintaining robust cybersecurity measures across all areas of operation. Overall results showed that there is indeed an impact of digital transformation on cybersecurity in this context, specifically in Gaza governorates, where it had an impact coefficient (0.897). Based on these findings, recommendations were made for improving electronic services offered by government agencies while also addressing gaps in worker performance related to technology use or knowledge gaps around best practices when dealing with sensitive information online. In conclusion: this paper provides valuable insights into how businesses can adapt their cybersecurity strategies when undergoing significant changes due to technological advancements such as those associated with “digital transformations”, identifying key organizational factors impacting cybersecurity measures across organizations like ministries.

Another study by Al Najjar et al. [42] aimed to identify the reality of digital transformation in the Palestinian Ministry of Interior and National Security from the point of view of workers in computer and information technology units. The study used a comprehensive survey method, distributing questionnaires among workers, with 61 retrieved (representing an 87.1% response rate). Several dimensions related to digital transformation were measured through these questionnaires, including senior management support, strategic directions, technical infrastructure necessary for digital transformation, human resources coordination, data privacy and security, organizational structure, and job description. The results showed that most dimensions related to digital transformation are available within the ministry to a large extent. However, there is still room for improvement, such as providing more funds for electronic services development or innovation spending. Senior management support received a high approval degree along with strategic directions. At the same time, the technical infrastructure necessary for digital transformation also achieved a large approval degree, followed by human resources coordination, which scored lower than other dimensions but still had significant relative weight. In conclusion, this paper highlights how important it is for organizations seeking competitive advantage through improved efficiency or low-cost electronic service growth opportunities that exploit technological revolution possibilities offered at all levels, internally or externally, with various partner institutions, to consider investing in their efforts toward achieving successful digital transformation initiatives.

In another study, Fjord and Schmidt [43] examined the potential and challenges of using digital tools to simplify tax assessment and collection and enhance transparency. Practical experiences in Denmark showed that states had made progress in making tax processes more efficient but needed to take measures to ensure legality and transparency through cybersecurity. 

Mijwil et al. [44] highlighted the importance of cybersecurity governance in digital transformation for public services provided by companies or institutions. The paper argued that changes in cybersecurity must be considered as it constitutes a large part of priorities for nations and companies undergoing digital transformation. The conclusion summarizes the importance of establishing straightforward programs and strategies to develop trustworthy cybersecurity governance without hacking or tampering with data/information while undergoing digital transformation. It also provided recommendations on how businesses can ensure secure operations while improving efficiency and effectiveness when providing public services through electronic means.

Maglaras et al. [45] focused on protecting critical infrastructure vital for public safety and national security. They proposed a methodology to protect critical national infrastructure based on fileless attacks versus Advanced Persistent Threat (APT) group techniques used in such attacks. The study using this methodology aimed to quantify and score cyber-attacks from an offensive cybersecurity perspective.

### 3.4. Business Sector

Business organizations are very heterogeneous, resulting in the technological systems deployed in the organizations. Modern-day technologies like the Internet of Everything can help organizations improve cybersecurity [46]. Gonchar [47] developed theoretical and practical recommendations for improving economic security in the digital economy. The researchers conducted a study on the impact of digital technologies on entrepreneurial activity in Ukraine, finding that businesses are increasingly using information and communication technologies. Still, there were differences based on size and sector. The paper proposed a methodology for assessing a country’s level of digital transformation within this context, which could help unify the study of conditions related to entrepreneurship and innovation. However, the paper found no significant relationship between the performance levels of the companies studied and their degree of digitization due to low staff involvement in these projects. The conclusion drawn from this research is that while businesses are adopting more technology across all sectors, including banking, as it increases flexibility and sales opportunities while decreasing costs incurred internally, such as time spent retraining employees who may not be familiarized yet or lack sufficient skills necessary at present given the rapid changes happening globally, there needs to be greater employee involvement in these projects if they are going to have an impact on business performance levels. Therefore, activities should focus not only on supporting enterprise resilience against risks associated with cybersecurity threats but also on promoting better employee qualifications required by more complex tasks resulting from business process automation through technology adoption across all sectors, including banking, where it increases flexibility and sales opportunities while decreasing costs incurred internally such as time spent retraining employees.

In another paper, Kuzior et al. [48] described the convergence of digitization processes across countries based on factors such as internet use, infrastructure metrics, and access to ICT. This study used the coefficient of variation to determine sigma convergence. It developed an econometric model that described the impact of national cybersecurity levels, ease of doing business, and anti-money laundering indices on digital development. This study aimed to understand the key determinants shaping risk in using financial instruments for money laundering and terrorist financing concerning global digitalization trends.

Moreover, another paper by Putri et al. [49] presented an example using the change from directory to digitization in Indonesia. Qualitative research approaches were used such as examining and describing events via interactions with others, mental images, and perceptions. These were drawn based on opinion from general public to encourage the use of digitization in public business and services and to follow trends observed from related parties as well as to encourage the government sector to develop services and evaluate the effectiveness of concepts using the six-ware cyber security framework (SWCSF) and Electronic-Based Government System (SPBE) that many government agencies have used. 

Furthermore, another study by Shitta-Bey [50] showed the impact of digital transformation through cloud computing on business transformation depending on the requirements factors chosen by organizations to publish or other models that differ from each other. The model and the scope of control were defined between cloud service providers and companion consumers. Therefore, there were security risks and broad threats associated with it, as well as an increase in the amount of confidential data in different cloud environments, and this is a significant concern for companies considering business transformation using a qualitative method to gain a thorough grasp of cloud computing service trends and practices. Among these threats to the cloud environment, whether from the inside or the outside, such as data penetration, loss, or leakage, dangers may also include weaknesses in the infrastructure or secure access, or they may be other destinations that are dead using the application programming interface. Eighteen threats were identified in this study of complete cloud migration. To deal with these security risks and take measures to reduce them and create strategies using appropriate equipment and recording procedures to monitor risks, suitable measures must be adopted during the migration or transition to the cloud. Protocols are included in the strategic plan that define the scope of migration and identify the basic parameters and indicators of performance.

E-commerce is an important application where digital transformation has transformed the business sector. Trung et al. [51] analyzed the applications of digital transformation, AI, IoTs, and blockchain in managing commerce secrets from a SWOT perspective. The authors used qualitative analysis, synthesis, inductive methods, and statistical data to conduct their research. They found that these technologies offer several benefits, such as increased efficiency, transparency, and security for businesses that adopt them. However, there are also challenges associated with their implementation, such as high costs and technical complexity, which need to be addressed by organizations before they can fully realize the potential benefits. In conclusion, the paper highlighted how mathematical solutions could be applied for industrial uses through a SWOT analysis of blockchain technology. It emphasized how businesses should consider adopting these technologies while being aware of their advantages and limitations to make informed decisions about implementing them into their operations effectively while minimizing cybersecurity risks in the industry 4.0 era or beyond.

Gul et al. [52] investigated Saudi E-commerce websites to understand the customers’ security perceptions using trustworthiness, credit card usage concerns, and consumer ratings as primary criteria. The authors found that Saudi E-commerce websites lack customers’ trust in the context of security, and there is a need to enhance the security features of Saudi websites. Similarly, Saeed [5] explored the user behavior of E-commerce customers in Pakistan using protection motivation theory as a theoretical model. The results highlighted that customer feelings, trustworthiness, motivation factors, and credit card concerns impact customer trust during online shopping.

### 3.5. Industrial Sector

Industry 5.0 advocates for establishing intelligent manufacturing systems, which require the Internet of Things based on technological implementation. There are many technological advancements to secure industrial organizations, such as automated attack detection [53,54], automated control rooms [55,56,57], zero trust architecture [58], and digital twins [59]. Osak and Buzina [60] explored ways to evaluate the flexibility and security of power systems under new conditions brought about by digital transformation and changes in industry practices, such as an increase in renewable energy sources and electric cars. The authors discussed principles for automatic control of power systems during digital transformation while considering differences between various electrical installations.

In another study, Mayhuasca and Sotelo [61] summarized how quantum technologies could revolutionize various industries by improving data processing capabilities and enhancing security against cyber threats. However, the authors acknowledged that further research is needed before these technologies can become widely adopted due to their complexity and the high cost currently associated with them. Overall, the authors suggested that continued exploration into quantum technology will likely lead to innovations that could transform our society even further than what we have seen with traditional computing systems.

In another study, Raza et al. [62] explored how organizations balance preventing security issues with responding to them in digital transformation projects. This research likely presents original insights into how organizations approach managing IS security compliance during digital transformation initiatives. This paper focused on Robotic Process Automation (RPA) in digital transformation and its impact on Information Security Compliance. Similarly, Trung et al. [63] explored how IoTs, machine learning (ML), AI, and digital transformation impact service industries such as education, medicine-hospitals, tourism, and manufacturing sectors. The authors found that in the education sector, ML and IoTs have affected teaching methods by evaluating students’ performance, which can help teachers choose suitable career development paths for learners. In the health sector, public health data processing is faster with big data due to ML technology being applied. Based on their empirical research findings, the authors proposed implications for future studies on applications of machine learning in each specific sector. They also highlighted cybersecurity risks associated with implementing these technologies that need management solutions. This study showed how emerging technologies like IoTs, machine learning (ML), and AI transform industries. Still, at the same time, it highlighted potential security risks associated with them, which need attention from researchers and practitioners who implement these systems into their organizations or businesses.

### 3.6. Diverse Organizational Contexts

In a study, Di et al. [64] proposed a networked organizational structure for enterprise information security management based on genetic algorithms and analyzed its benefits compared to traditional approaches. The authors identified the challenges enterprises face in managing their information security during digital transformation efforts, such as risks from cyber-attacks and data breaches. They proposed a new genetic algorithm approach to improve work efficiency, reduce costs, and maintain strong information security. Their experiments comparing traditional network organization structures with those based on genetic algorithms found that the latter was much more efficient in terms of work efficiency. Additionally, they provided data showing advantages such as cost savings and room for growth when implementing this approach within enterprises. Overall, the results suggested that using a networked organizational structure for enterprise information security management based on digital transformation and genetic algorithms can effectively maintain strong information security while improving work efficiency within businesses undergoing technological change.

Alenezi [65] examined the role of software engineering in digital transformation and its importance for secure development practices. The authors argued that software engineering has become essential in ensuring efficient functioning as organizations increasingly adopt digital solutions to improve their operations. They also highlighted that security concerns are critical during this process due to increased cyber threats. Analyzing trends in software engineering and examining case studies from various industries, such as healthcare and finance, they conclude that all digital systems rely on software for efficient performance while emphasizing how secure development practices can mitigate risks associated with adopting new technologies.

Moreover, in another paper, Marelli [66] discussed how digitization and new technologies are becoming increasingly crucial in humanitarian operations, making organizations vulnerable to cyber-attacks that can impact their ability to protect and assist those affected by armed conflict and violence.

In another study, Dvojmoč and Verboten [67] emphasized that employers have certain obligations to ensure employee information security, such as using appropriate hardware and software, configuring firewalls, and implementing antivirus programs. Furthermore, they highlighted the need for companies to comply with international instruments such as the GDPR when dealing with personal data protection issues related to new technologies being implemented. 

On the other hand, in the environmental sector, Mukhlynina et al. [68] examined the problem of introducing digital technologies into the system of environmental safety and protection in Russia. The authors focused on the role and specific steps currently being taken by state authorities at the federal level. They also highlighted legal problems that exist in this context. The detailed findings suggested several challenges associated with implementing digital transformation efforts related to environmental safety in Russia. These included a lack of clear regulatory frameworks, insufficient funding for research and development activities, inadequate infrastructure support, and limited public awareness about these issues. In terms of results, based on their analysis using the factor analysis method, they identified vital factors affecting digitization efforts, such as technological readiness, availability of a skilled workforce, government policies and regulations, etc., which can be used by policymakers while designing strategies toward achieving sustainable environmental goals through digitization. Furthermore, Halabi et al. advocated for green cybersecurity practices to save energy consumption [69].

Voskresenskaya [70] investigated the current state of digital transformation in governance, economy, and social sectors as a factor for development and security. The researchers found that digitalization has become an integral part of modern society. They identified vital attributes such as the mechanism for transforming economic cooperation into information/telecommunication space, active introduction/application of e-money/smart contracts into civil transactions, and development of e-governance. They also noted that problems within these areas could affect compatibility with other economies due to lagging data processing capabilities or the inability to use digital resources effectively. Based on their analysis using both qualitative (laws/regulations) and quantitative (statistical/comparative) methods at national/international levels, they concluded that there are significant benefits associated with embracing digitization across various sectors, including increased efficiency/productivity in service delivery processes, which ultimately leads toward sustainable growth/security.

In conclusion, it was recommended that governments prioritize investment in infrastructure necessary for the effective implementation/adoption of new technologies while ensuring that adequate regulation/policy frameworks exist to support innovation without compromising citizens’ privacy/data protection rights. Additionally, given the rapid pace of change, businesses must adapt quickly to remain competitive. In another study, Kuchumov et al. [71] suggested that while there are potential benefits from digitization initiatives, such as increased efficiency and productivity gains, significant risks are involved, such as cybersecurity threats or job displacement due to automation. Furthermore, the impact of these initiatives varies depending on regional policies toward digitization. In conclusion, this paper highlighted that it is essential that policymakers in Russia’s regions consider potential benefits and carefully evaluate possible negative impacts when implementing digital transformation strategies. By doing so, they can develop adequate public policies based on systemic analyses that take into account both positive effects along with serious risk factors affecting further development within each region individually rather than applying one-size-fits-all solutions across all areas indiscriminately without considering local conditions or needs specificities, which could lead to unintended consequences if not adequately addressed beforehand through careful planning processes involving stakeholders at different levels (local communities/businesses/government agencies).

Alahmadi et al. [72] highlighted that digital agriculture has helped automate labor-intensive jobs. However, many threats and vulnerabilities are associated with digital agriculture. They highlighted the potential side-channel attacks relevant to digital transformation. Similarly, Song et al. [73] highlighted that the Internet of Things and 5G networks have resulted in massive growth of digital agriculture. However, publishing a large volume of data is prone to security concerns. As a result, the authors have proposed a privacy-preserving data aggregation scheme that is more secure and flexible.

Gonçalves [74] highlighted that digital transformation in the accounting sector of small- and medium-scale enterprises is in its early stages; however, the benefits are widely recognized. Data protection and cybersecurity threats are vital challenges that need to be handled by accounting professionals. In another study, Tiron-Tudor et al. [75] highlighted that artificial intelligence, blockchain, and GPS technologies can help companies’ accounting departments implement real-time auditing systems. However, companies must allocate substantial resources to mitigate cybersecurity risks associated with advanced technologies.

Rodríguez-Abitia and Bribiesca-Correa [76] highlighted the fact that technological advancements, such as artificial intelligence, the Internet of Things, blockchain, 3D printing, and secure technical infrastructure, will also change universities. Everyone may adopt a new role, such as content producer, influencer, etc., to contribute to the education sector. Similarly, Pavlova [77] highlighted that the culture is typically based on free and open knowledge sharing in an educational setting. However, security threats demand a balance between openness and security mechanisms. Table 1 provides a summary of all the literature discussed.

Power systems are complex infrastructures in modern society and are vulnerable to cybersecurity threats [78,79]. Dagoumas [80] has used IEEE RTS 96 power system, and the author highlighted that a combination of operating conditions and cyber-attacks should be used to evaluate system stability. Diaba et al. [81] highlighted that power system communication protocols are prone to cyber-attacks by hackers. The authors have proposed an algorithm outperforming conventional deep learning approaches using SVM, ANN, and CNN. Similarly, Presekal et al. [82] developed a hybrid machine learning model using Graph Convolutional Long Short-Term Memory (GC-LSTM) and a deep convolutional network for anomaly detection in electrical power grids.

Kechagias et al. [83] highlighted that cybersecurity in the maritime industry has become very important. The authors have presented a detailed case of how a maritime company adopted a systematic approach to review its cybersecurity strategic policies, found loopholes, and subsequently performed risk mitigation.

**Table 1:** Key findings of literature.

Paper	Publication Year	Application Domain	Key Technologies/Theories	Key Findings
[22]	2022	Financial sector	Artificial intelligence, cognitive mapping, DEMATEL techniques	Provision of a decision-support model by combining the decision-making trial and evaluation laboratory (DEMATEL) method and cognitive mapping.
[23]	2023	Financial sector	Cognitive technologies	Provided directions to use cognitive technologies in the digital transformation of the Russian economy.
[24]	2022	Financial sector	Artificial intelligence, blockchain, voice-based technology, or natural language processing	Higher trust in Fintech by stakeholders in the financial sector.
[25]	2019	Public institutions, financial institutions, banking institutions, industry, transportation, and agriculture	Different technologies related to digitalization	Highlighting the need for information security in different application domains.
[34]	2023	Healthcare sector	No specific technology was mentioned	Sustainability of digital transformation in the healthcare sector requires cybersecurity skills, uncertainty management, and the healthcare sector’s interdependence.
[35]	2023	Healthcare sector	Electronic health records, remote patient monitoring, artificial intelligence, telemedicine, and federated learning	Privacy and security recommendations for the healthcare sector.
[36]	2020	Healthcare sector	Smartphone apps and wearable tech products enable data sharing	Need for data privacy of patients in healthcare applications.
[37]	2021	Healthcare sector	No specific technology was mentioned	Need for enhanced cybersecurity in post-COVID-19 digital transformation.
[41]	2022	Government sector	No specific technology was mentioned	Recommendation to use a secure network by the ministry in Palestine.
[42]	2022	Government sector	No specific technology was mentioned	Establishment of clear data exchange policies and clear job descriptions for IT employees.
[43]	2023	Government tax payments	Full-service mobile apps and e-payment channel	Need for actions to make the process transparent and legal in Danish tax payment.
[44]	2023	Governmental and other public services	AI and other leading technologies	Emphasizes cybersecurity governance.
[45]	2021	Government critical infrastructure	No specific technology was mentioned	Emphasizes cybersecurity of critical infrastructure.
[47]	2022	Business sector	No specific technology was mentioned.	Emphasizes state regulations for the transformation of economic clusters at the international level.
[48]	2022	Business sector	Advanced encryption and data analytics are essential for cybersecurity and AML efficiency	Analyzed digital transformation and cybersecurity situations across different countries.
[49]	2022	Business sector	Business sector advanced encryption and data analytics are essential for cybersecurity and AML efficiency	Digital transformation in Indonesia and six-ware cyber security framework.
[50]	2023	Business sector	IT security and data protection, cloud migration, cloud computing	Security concerns for cloud transformation of business.
[51]	2021	Business sector	Blockchain, IoTs, AI, and other emerging technologies	SWOT analysis of blockchain and other technologies.
[52]	2022	E-commerce	Trustworthiness, credit card usage concerns, consumer rating	User information security perception in Saudi Arabian E-commerce applications.
[5]	2023	E-commerce	Protection Motivation Theory	User information security perception of E-commerce in Pakistan.
[60]	2023	Industrial sector	No specific technology was mentioned	Security implication of small power plants.
[61]	2022	Industrial sector	Quantum computing, cryptography, optical fiber, and related technologies are discussed	Information security implications in quantum technologies.
[62]	2019	Industrial sector	Robotic Process Automation (RPA)	Information system security compliance and response implications.
[63]	2019	Service industry	IoTs, machine learning, AI, and digital transformation	Cybersecurity implications of IoTs, machine learning, and digital transformation.
[64]	2022	Networked organizational structure	Genetic algorithms	Factors affecting quality in manufacturing settings.
[65]	2021	Work environment	Cloud computing	Emphasizes the importance of secure software development in digital transformation.
[66]	2020	Enterprises	Mobile devices, cloud computing, social media platforms	Cybersecurity implications for the digital transformation of humanitarian organizations.
[67]	2022	Work environment	Firewalls, encryption software, intrusion detection systems	Emphasizes data security of employee data in organizations.
[68]	2022	Environment	No specific technology focused on	Digital transformation and environmental security in Russia.
[69]	2022	Environment	IoTs	Green IoTs and adaptive cybersecurity implications.
[70]	2019	Governance, economy, and social sectors	No specific technology was mentioned	Digital transformation and security in Russia.
[71]	2020	Economy	No specific technology was mentioned	Economic security and digital transformation in Russia.
[72]	2022	Digital agriculture	Smart sensors, Internet of Things, machine learning	Side-channel attacks in digital agriculture.
[73]	2020	Digital agriculture	Internet of Things, 5G networks	Privacy-preserving data aggregation scheme.
[74]	2022	Digital accounting	Robotics, enterprise resource planning, artificial intelligence, optical character recognition	Digital transformation and future of accounting.
[75]	2022	Digital accounting	AI, blockchain, cloud computing	Emphasis on benefits for accounting firms in digital transformation.
[76]	2021	Education	Artificial intelligence, Internet of Things, blockchain, 3D printing, cybersecurity, big data	Futuristic universities in the era of digital transformation.
[77]	2022	Education	No specific technology focused on	Emphasis on cybersecurity culture in universities.
[80]	2019	Power systems	IEEE RTS 96	Emphasizes the impact of cybersecurity attacks on power systems.
[81]	2023	Power systems	Artificial neural networks, convolutional neural networks, and support vector machines	Meta-heuristic and deep learning algorithms for cybersecurity in power systems.
[82]	2023	Power systems	Graph Convolutional Long Short-Term Memory (GC-LSTM) and a deep convolutional network	Model for situational awareness in online cyber-attack.
[83]	2022	Maritime	No specific technology focused on	Cybersecurity implications for maritime industry.

## 4. Discussion

**Figure 3:**
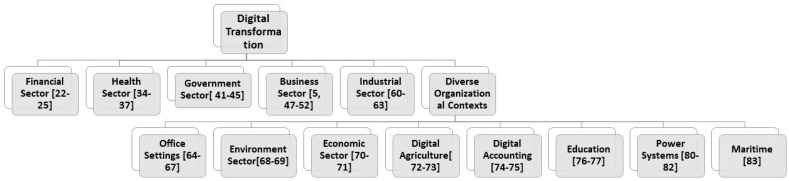
Taxonomy of the literature.

Table 1 highlights that advanced technologies such as the IoTs [51,53], blockchain [63,74], and 5G [84,85] networks can facilitate organizations in securing business processes and making them efficient [1,2]. Furthermore, machine learning approaches [63,72,86] can help identify malicious traffic in the network, which can help in identifying cyber threats proactively. However, such technological interventions should be well thought out and appropriately designed [9]. While new technologies can increase efficiency and competitiveness of businesses, they also bring unknown risks, such as cyber-attacks [3]. This leaves them vulnerable to cyber threats, which could have significant economic consequences. Therefore, raising awareness about these risks among industry professionals is essential. Additionally, there should be reasonable security measures to secure technological infrastructures from cyber-attacks [87]. A fundamental security strategy could help organizations from recurring cyber-attacks [88]. Therefore, it is important to analyze cybersecurity risks during the transition to the digital economy [89]. Governments have an extensive role in developing and implementing national-level policy. For instance, establishing a national cybersecurity strategy has helped Greece pursue digital transformation [90]. It should also be considered that while aiming for digital transformation, human factors should also be considered. Human performance degradation is a critical factor in cybersecurity attacks [91]. As shown in the taxonomy of the literature in Figure 3, every sector of the economy is benefiting from the advances in digital transformation and trying to minimize cybersecurity risks.

**Figure 4:**
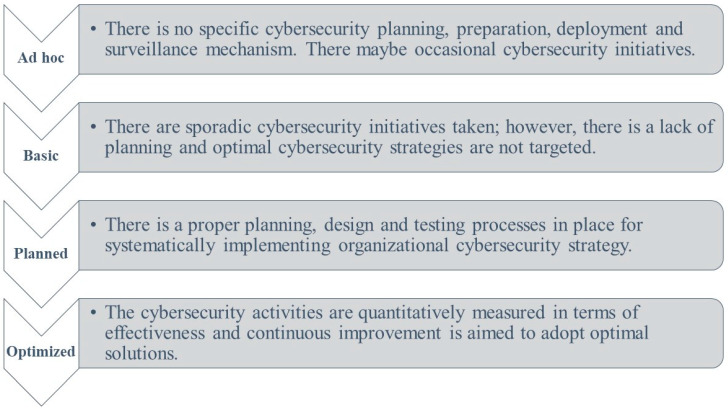
Cybersecurity readiness framework for business organizations.

In the literature, some review articles have focused on digital transformation, such as an article by Metawa et al. [92], which investigated the role of information in digital transformation in the context of Egyptian small- and medium-scale enterprises. Similarly, Özsungur [93] researched business strategy for cybersecurity in digital transformation, and Nguyen Duc [94] documented security risk from an engineering perspective. Furthermore, Hai et al. [1] highlighted the opportunities and challenges for emerging countries regarding digital transformation, and Kour’s work [95] focused on cybersecurity implications in the railway domain. Despite these surveys within the literature, no survey has presented a domain taxonomy and looked into cybersecurity implications in diverse industries, as has been explored in this paper. Based on our review, we propose a cybersecurity readiness framework for business organizations pursuing digital transformation. As shown in Figure 4, this framework has four levels.

At the ad hoc level, organizations do not have planning, preparation, deployment, and surveillance mechanisms to respond to cybersecurity threats. Cybersecurity resilience is dependent on the personal initiatives of employees. Emerging technologies such as artificial intelligence, big data and analytics, blockchain, cloud computing, and services drive digital transformation worldwide while increasing cybersecurity risks for businesses undergoing this process. Therefore, it is crucial to analyze cybersecurity measures during implementation in pursuit of digital transformation, but the organizations at this level do not focus on these aspects.

At the basic level of our framework, organizations have essential cybersecurity planning, preparation, deployment, and surveillance activities in place but no organizational strategic policy regarding cybersecurity. The processes are not mature, and isolated efforts are carried out; no data about the effectiveness of employed cybersecurity approaches are available.

At the planned level of our framework, organizations need a well-planned organizational cybersecurity strategy documenting the processes for cybersecurity preparation, deployment, and surveillance. During the surveillance phase, potential vulnerabilities must be regularly assessed through penetration testing or vulnerability scanning. In addition, it is essential for organizations undergoing DT to consider the human factor in cybersecurity. This means providing regular training and awareness programs for employees to identify and respond appropriately to potential cyber threats. Furthermore, as technology advances rapidly, new security risks not yet fully understood or addressed by current security measures will likely emerge. It will be crucial for businesses undergoing DT involving IoTs devices or other emerging technologies like 5G networks or quantum computing to prioritize comprehensive risk assessments before implementing such solutions. 

Organizations aiming for an optimized level need to continuously measure the effectiveness of their cybersecurity planning, preparation, deployment, and surveillance mechanisms. As technology evolves rapidly and new cyber threats constantly emerge, vulnerabilities may arise even with robust security measures. Therefore, it is essential for organizations undergoing DT to perform futuristic technological forecasting and associated cybersecurity planning to continuously innovate their processes. A proactive approach toward optimized security processes can help mitigate future risks associated with digital transformation efforts. 

## 5. Conclusions

This systematic literature review has shed light on the critical role of cybersecurity in digital transformation (DT). Digital transformation has transformed the business sector by transitioning organizational processes to IT solutions, resulting in significant changes across various aspects of an organization. It impacts multiple elements, such as user experience, operations, markets, customers, relationships, and cultural differences. Emerging technologies, including artificial intelligence (AI), big data and analytics, blockchain technology, cloud computing, and services, drive digital transformation worldwide while increasing cybersecurity risks for businesses undergoing this process. And the implications of cybersecurity for digital transformation are significant. As enterprises undergo the process of digital transformation, they become more vulnerable to cyber-attacks and security breaches. Cybersecurity is an essential component of digital transformation as it helps prevent interruptions due to malicious activities or unauthorized access by attackers aiming at sensitive information alteration, destruction, or extortion from users. The COVID-19 pandemic has further highlighted the importance of cybersecurity in DT implementation, as cybercriminals have taken advantage of vulnerabilities created by this rapid shift toward digitalization. Therefore, organizations undergoing DT adoption must prioritize cybersecurity measures to ensure a successful transition without any disruptions caused by security breaches. The study highlights that DT is a complex and ongoing process that requires organizations to be aware of emerging technologies and their associated security risks. As businesses transition their primary operations to IT solutions, they must ensure appropriate measures are in place to protect data and networks from unauthorized access or malicious activities. The findings suggest that implementing encryption or cyber insurance policies can help mitigate these risks during DT implementation. For future studies, we recommend the importance of organizations having comprehensive knowledge of cybersecurity threats throughout the entire process. This includes identifying potential vulnerabilities early on and proactively addressing them.

## Data Availability

Not applicable.
